# The Number and Structure of Muscle Synergies Depend on the Number of Recorded Muscles: A Pilot Simulation Study with OpenSim

**DOI:** 10.3390/s22228584

**Published:** 2022-11-08

**Authors:** Cristina Brambilla, Alessandro Scano

**Affiliations:** 1 Institute of Intelligent Industrial Systems and Technologies for Advanced Manufacturing (STIIMA), Italian Council of National Research (CNR), 23900 Lecco, Italy; 2 Institute of Intelligent Industrial Systems and Technologies for Advanced Manufacturing (STIIMA), Italian Council of National Research (CNR), 20133 Milan, Italy

**Keywords:** muscle synergies, OpenSim, EMG, upper limb

## Abstract

The muscle synergy approach is used to evaluate motor control and to quantitatively determine the number and structure of the modules underlying movement. In experimental studies regarding the upper limb, typically 8 to 16 EMG probes are used depending on the application, although the number of muscles involved in motor generation is higher. Therefore, the number of motor modules may be underestimated and the structure altered with the standard spatial synergy model based on the non-negative matrix factorization (NMF). In this study, we compared the number and structure of muscle synergies when considering 12 muscles (an “average” condition that represents previous studies) and 32 muscles of the upper limb, also including multiple muscle heads and deep muscles. First, we estimated the muscle activations with an upper-limb model in OpenSim using data from multi-directional reaching movements acquired in experimental sessions; then, spatial synergies were extracted from EMG activations from 12 muscles and from 32 muscles and their structures were compared. Finally, we compared muscle synergies obtained from OpenSim and from real experimental EMG signals to assess the reliability of the results. Interestingly, we found that on average, an additional synergy is needed to reconstruct the same *R*^2^ level with 32 muscles with respect to 12 muscles; synergies have a very similar structure, although muscles with comparable physiological functions were added to the synergies extracted with 12 muscles. The additional synergies, instead, captured patterns that could not be identified with only 12 muscles. We concluded that current studies may slightly underestimate the number of controlled synergies, even though the main structure of synergies is not modified when adding more muscles. We also show that EMG activations estimated with OpenSim are in partial (but not complete) agreement with experimental recordings. These findings may have significative implications for motor control and clinical studies.

## 1. Introduction

**Figure 1:**
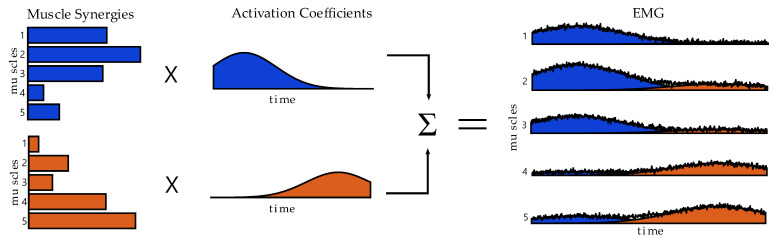
**The spatial synergy model**. The spatial synergy model decomposes the EMG signal in spatial muscle synergies and activation coefficients. The linear summation of the synergies and of the corresponding activations allow reconstruction of the EMG signals.

The central nervous system (CNS) coordinates a large set of joints and muscles in order to perform specific and complex tasks in the workspace. To achieve this, it has been hypothesized that the CNS simplifies motor control combining a set of modules, also called muscle synergies [1]. The muscle synergy model has been used to evaluate motor control in several fields of research and it represents the state-of-the-art approach for quantitatively determining the modules that the CNS uses to simplify the planning and the execution of the movement [2]. The spatial synergy model is based on the hypothesis that the processed electromyographic (EMG) signal can be decomposed in muscles synergies, that are invariant across several classes of movements and can be modulated by variant temporal coefficients to adapt to specific tasks [1]. The muscle synergy analysis is an attractive framework that allows to quantitatively assesses control structures and was employed in a variety of motor control [1,3], rehabilitation [4,5], gait analysis studies [6,7], and others. Figure 1, inspired from Cheung at al. [4], depicts the spatial synergy model in a synthetic way.

According to the spatial model, the number of the muscle synergies extracted represents the dimensionality of the motor control space for the classes of analyzed movements. Therefore, the choice of the number of EMG channels may influence the resulting number of motor modules. In experimental studies, typically 8 to 16 EMG channels, and always below 20, are used depending on the application. For the upper limb, four to five synergies were identified by d’Avella et al. [8] from 19 muscles. Scano et al. [9] employed 14 EMG signals in a monolateral set-up extracting 3 to 4 synergies that reconstructed 85% of the original signal. The same dimensionality of the control space was found in Zhao et al. [10] when analyzing 16 muscles. Russo et al. [11] found five synergies using 18 EMG probes in a similar set-up based on upper-limb movements. During locomotion, a set of four synergies were identified from 13 recorded muscles [12]. Delis et al. [13] recorded 30 muscles simultaneously, but they were distributed in a total body configuration. The use of a limited number of muscles in synergy analysis may underestimate the real dimensionality of the control space, leading to significative implications for motor control and clinical studies.

A dense mapping of muscles from the same anatomical sector (e.g., the upper limb) can give a more detailed description of muscle activations and their role in movement execution. However, there are some limitations that prevent the applicability of high-density EMG recordings. Usually, the selected muscles are superficial; moreover, only large muscles that are easily detectable and generate clearly distinguishable EMG signals are recorded. Some muscles are often not considered since they have deep muscle bellies, or they are too small, and are subject to cross-talk. The amplitude of the signal registered from a deep muscle is low in magnitude and noisy due to the thickness of the tissue between the muscle and the probe [14]. Moreover, the EMG signal detected over a non-active muscle and generated by a nearby muscle may generate cross-talk [15], and, therefore, recording the activity of some muscles may lead to incorrect interpretation of the data if ad hoc set-ups are not arranged to minimize cross-talk. Lastly, using more probes requires expensive equipment and is not always compatible with the time available for clinical exams.

Therefore, implementing experimental approaches for the recording of the EMG signals from a large set of muscles is challenging. In this context, musculoskeletal simulations can be used to estimate the muscle activations starting from experimental kinematic data. In particular, OpenSim is an open-source, well documented software that allows dynamic simulations with musculoskeletal models [16]. Musculoskeletal models have been used already for various biomechanical and motor control studies. These models allow to simulate various conditions, to assess the outcomes of kinematics and biomechanics, and the estimation of muscle activations. In the lower-limb, the effects of walking speed on joints and muscles [17] or the difference between walking barefoot or with shoes [18] was assessed with musculoskeletal models. Moreover, the difference in muscle strength between healthy subjects and patients, as diabetics [19], or elderly subjects [20], was assessed. Musculoskeletal simulations were also used for postural analysis in working environments [21,22]. Muscle synergies have also been extracted from simulated muscle activations from OpenSim during isometric tasks by Steele et al. [23], which considered different subsets of all the muscles for synergy extraction.

In this paper, we investigated if considering a comprehensive set of muscles, the muscle synergies extracted are different in number and structure. Since the musculoskeletal models represent valuable tools to estimate conditions that cannot be easily assessed with experimental approaches, we estimated the activations of a large set of muscles with an upper-limb musculoskeletal model implemented in OpenSim. We simulated a set of multi-joint upper-limb movements using experimental data [24]; then, we extracted spatial synergies from a 32 muscles model and compared it with a standard 12 muscles model when reconstructing the same *R*^2^. In particular, we analyzed if the additional muscles were included in synergies with muscles that have similar functions or if they generated new synergies.

The aim of this work was to compare the number and structure of synergies when comparing a standard multi-channel EMG set-up with a high-density mapping. This work extends previous findings [23] by proposing the following novel analyses: the use of EMG data from paradigmatic dynamic movements taken from experimental laboratory trials; the separation and use of the phasic EMG components (movement-related) for the extraction of muscle synergies, with removal of the tonic EMG components (anti-gravity); the inclusion of a high number of upper-limb muscles that move the forearm and the wrist. Finally, we compared the muscle synergies extracted from the activations estimated with OpenSim and the ones obtained from real experimental EMG signals recorded in a laboratory environment in order to assess the reliability of the results of the simulation.

## 2. Materials and Methods

**Figure 2:**
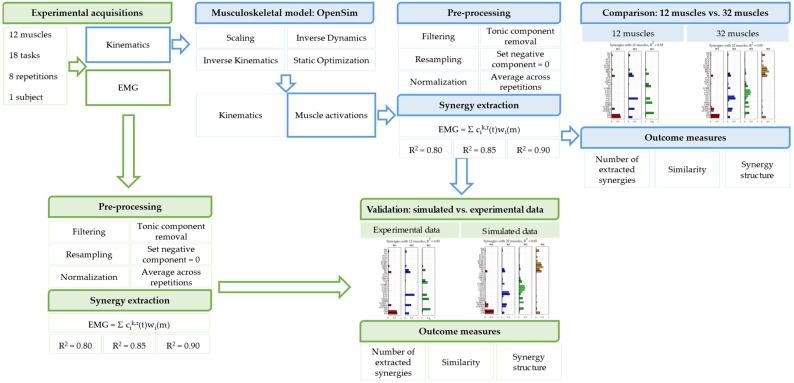
**Scheme of the work**. Experimental kinematic data were used to compute the inverse kinematics and the muscle activations in a musculoskeletal model in OpenSim. Synergies were extracted from the resulting muscle activations, with a 12 muscles model and a 32 muscles model; the extracted synergies were compared between the two conditions. Synergies were also extracted from experimental EMG data, and the results were compared to the synergies extracted from simulated data in order to assess the reliability of the simulated approach.

The structure of the work is portrayed in Figure 2 with a scheme.

### 2.1. Participants

Data from one subject were at the basis of our simulation. The subject recruited to participate in this study (35 years old; 81 kg; 1.80 m) had no musculoskeletal impairment affecting performance. Ethical approval was granted by the CNR Ethical Committee (Rome, Italy) and the experimental trial was conducted in compliance with the Declaration of Helsinki [25]. The participant provided written informed consent to participate in this study and for the analysis of any data included in this article.

### 2.2. Laboratory Acquisition Set-Up

**Figure 3:**
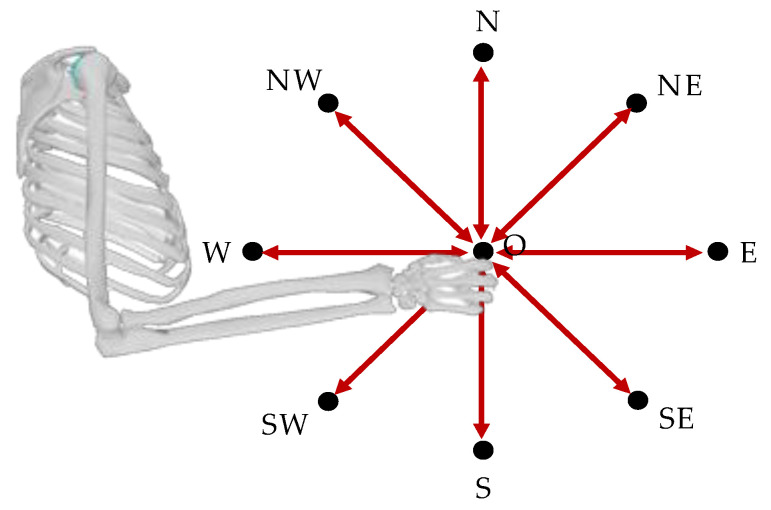
**Acquisition set-up**. The target board was oriented in sagittal plane and the subject performed movements with the right arm (center-out movements, e.g., 0 - > N, and out-center movements, e.g., N - > 0.

Kinematic data were acquired using the Vero Vicon system, a marker-based tracking system consisting of 10 infrared cameras and a set of 25 retroreflective markers placed at anatomical landmarks as in the Vicon Upper-Limb model protocol. The subject stood in the middle of the area tracked by the motion capture system and executed a set of multi-directional point-to-point movements toward 9 targets indicated by markers placed on a target board at 8 cardinal points (NE, E, SE, S, SW, W, NW, N) and in the center of a circle (labelled as O), as in previous similar protocols [8,9]. Movements were performed in forward (center-out e.g., N -> 0;) and backward direction (out-center e.g., 0 -> N) for a total of 16 movements per trial. The subject was asked to perform the movement very fast, so that the maximum duration of each movement was lower than 0.4–0.5 s [8,9], to elicit phasic (motion-related) EMG activity; the subject had to wait a second in static condition at each target before moving to the following one. The target board was oriented in the sagittal plane with respect to the subject and the movement was executed with the right upper limb, as shown in Figure 3. The trial was repeated ten times to increase the signal-to-noise ratio (SNR).

The EMG signals were recorded using 12 bipolar electrodes (Cometa, Italy) positioned according to SENIAM guidelines [26] on 12 muscles of the right upper limb: teres major (TM), infraspinatus (IF), deltoid anterior (DA), deltoid middle (DM), deltoid posterior (DP), pectoralis (PT), triceps long head (TL), triceps lateral head (TLa), biceps long head (BL), biceps short head (BS), pronator teres (PT) and brachioradialis (BR).

### 2.3. Data Analysis

#### 2.3.1. Experimental Data Analysis

Kinematic data were filtered with a 3rd order Butterworth lowpass filter with cut-off frequency = 6 Hz. The trajectory of the wrist marker was used to identify movement phases: the onset and the offset of the movements were considered when 5% of the maximum absolute velocity of the wrist was exceeded.

#### 2.3.2. OpenSim Model

**Figure 4:**
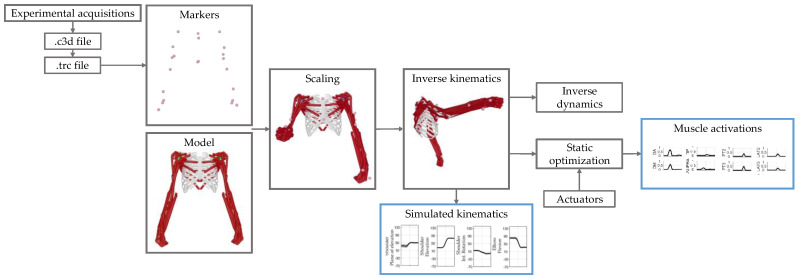
**Workflow for data analysis in OpenSim and Matlab.** Marker trajectories were exported from experimental acquisitions, in order to be imported in OpenSim. The musculoskeletal model was scaled and the inverse kinematics was computed, obtaining the simulated kinematics. The inverse dynamics and the static optimization were computed based on the inverse kinematics results, in order to estimate the muscle activations.

The upper-limb musculoskeletal model was available in OpenSim [27] and allowed computation of the kinematics, dynamics and activations of muscles. The model is a bimanual upper-limb model with 22 rigid bodies and 20 degrees of freedom: three translations and three rotations for the trunk, plane of elevation, elevation and internal/external rotation of the shoulder, elbow flexion/extension and pronation/supination, flexion and ulnar deviation of the wrist. The model is actuated with 100 muscles, fifty for each arm. Simulations and analyses were performed in OpenSim 4.4; the workflow employed in the OpenSim environment is schematized in Figure 4. First, the model was scaled based on the marker locations in a static pose, and, then, the scaled model was used for the inverse kinematics and inverse dynamics computation. The 3D marker trajectories were converted in a .trc file and they were given as input to OpenSim in order to determine the kinematics and the dynamics of the movement. Muscle forces were computed with the static optimization procedure provided in OpenSim [28], in which the muscle forces are calculated minimizing the instantaneous total square muscle activation needed to achieve the experimentally-acquired trajectory. The model includes the muscle force–length–velocity relationships. Since the total muscle moment was insufficient to balance the joint moment, reserve actuators were added to stabilize the model, as suggested in OpenSim tutorials and in previous work [29].

The resulting kinematics and muscle activations were imported in Matlab for further elaboration. Kinematic data were filtered with a 3rd order Butterworth low pass filter with cut-off frequency = 6 Hz. The considered muscles were those of the right (dominant) upper limb, excluding the trunk and hand muscles, and were divided in the “standard” dataset of 12 muscles and the “high-density” dataset of 32 muscles. The standard dataset was composed of the following muscles: deltoid anterior (DA), deltoid middle (DM), deltoid posterior (DP), infraspinatus (INFRA), teres major (TMAJ), pectoralis major sternal (PT2), triceps long (TL), triceps lateral (TLat), biceps long (BL), biceps short (BS), brachioradialis (BRD) and pronator teres (PT). The “standard” dataset includes muscles frequently recorded in most of the experimental studies. In the “high-density” dataset, the following muscles were added to the previous 12: supraspinatus (SUPRA), subscapularis (SUBSC), teres minor (TMIN), pectoralis major clavicular (PT1), pectoralis major ribs (PT3), latissimus dorsi thoracic (LAT1), latissimus dorsi lumbar (LAT2), latissimus dorsi iliac (LAT3), coracobrachialis (CORB), triceps medial (TMed), anconeus (ANC), supinator (SUP), brachialis (BRA), extensor carpi radialis longus (ECRL), extensor carpi radialis brevis (ECRB), extensor carpi ulnaris (ECU), flexor carpi radialis (FCR), flexor carpi ulnaris (FCU), palmaris longus (PL) and pronator quadratus (PQ). The “high-density” dataset includes muscles frequently not recorded.

#### 2.3.3. Simulated and Experimental EMG Pre-Processing

The simulated muscle activations were filtered with a 7th order Butterworth filter with cut-off frequency = 10 Hz. The considered movement phases began 0.3 s before the kinematic onset and 0.3 s after the kinematic offset, in order to detect complete muscle activation waveforms that could begin before kinematic movement due to electromechanical delay and muscle pre-activation [30]. Since the muscle activations contain a phasic component related to the movement and a tonic component with an anti-gravity and stabilizing function [31], the tonic component was modelled with a linear ramp and removed from the muscle activation envelopes to achieve phasic muscle activation envelopes, as in previous studies of the field [8]. Lastly, minor negative components originated with the EMG components’ subtraction were set to zero in order to use the non-negative matrix factorization (NMF) algorithm. Each movement phase was resampled at 100 samples and the eight repetitions were averaged. In order to guarantee inter-acquisition and inter-muscle data comparison, each muscle activation was normalized to the maximum value of all the muscles across all the trials.

The experimental EMG signals were filtered with a 7th order Butterworth high pass filtered with a cut-off frequency of 30 Hz, rectified and low pass filtered at 10 Hz. Then, the same pipeline used to compute muscle activations used for simulated data was performed. Therefore, in each movement phase, the tonic components were removed from the EMG signal and the negative components were set to zero. Then, each movement phase was resampled at 100 samples and the repetitions were averaged. Each EMG signal was normalized to the maximum value of all the EMG signals across all the trials.

#### 2.3.4. Synergy Extraction

Considering M as the number of muscles and K as the number of movement phases, the activations were pooled in a matrix with M rows and K·100 columns for each repetition. The eight repetitions were averaged before synergy extraction. The NMF algorithm [32] was used to extract spatial synergies

$$EMG\left(t,k,m\right)=\overset{S}{\underset{i=1}{\sum }}c_{i}{}^{k}\left(t\right)\;w_{i}\left(m\right)$$

where $w_{i}$ are the time-invariant synergy vectors and $c_{i}$ the time-varying scalar activation coefficients for each synergy (*i* = 1…S), and $EMG\left(t,k,m\right)$ the activity of muscle *m* at time *t* of task *k*. Synergy extraction leads to *S* synergies (each a column vector with N components) and S×K time-varying coefficients (100 samples each). Each spatial synergy was normalized by its Euclidean norm and, as a consequence, synergies had unit norm and the temporal coefficients were also normalized by the reciprocal of the norm to preserve the magnitude of the reconstructed signal. The goodness of reconstruction was evaluated with the $R^{2}$ defined as $1-\frac{SSE}{SST}$ where *SSE* is the sum of the squared residuals and *SST* is the sum of the squared differences with the mean EMG vector [8]. Synergies were extracted from order 1 to order N (number of muscles) and the algorithm was applied 200 times in order to avoid local minima. The repetition with the highest *R*^2^ was chosen as the representative for that order.

### 2.4. Outcome Measures and Statistical Analysis

The goodness of the reconstruction *R*^2^ was computed when considering muscle activations in the two OpenSim cases: 12 muscles and 32 muscles. Synergies were extracted according to three levels of minimal reconstruction *R*^2^—0.80, 0.85 and 0.9—usually used in the literature [33]. For each *R*^2^ threshold, the order of factorization and the synergy structure were compared. First, synergies extracted in the two conditions were matched in pairs by similarity. The cosine angle computed between matching synergies was the measure of similarity; the similarity was computed only on the 12 muscle loads common to the two conditions. To guarantee to operate on unit norm synergy loads, the subset of 12 used loads in the 32-muscles case was rescaled to unit norm before computing similarity.

The same procedure was used to compare the synergies extracted from the simulated data and the experimental EMG data.

## 3. Results

### 3.1. Simulation Results

**Figure 5:**
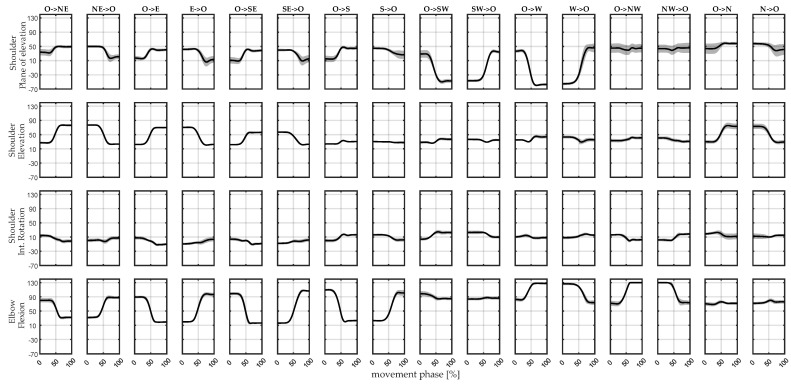
**Simulated kinematics**. Kinematics obtained from OpenSim simulations during each direction of motion. The bold lines represent the mean of the repetitions and the shaded areas are the standard deviations. From top to down, shoulder plane of elevation, elevation and internal rotation and elbow flexion are reported in rows; the directions of movement are reported in columns.

The resulting kinematics obtained from the simulations in OpenSim are reported for each direction of movement, after averaging the trial repetitions, in Figure 5. We reported the three degrees of freedom of the shoulder and the elbow flexion of the right arm.

The maximum range of motion of the shoulder plane of elevation was 119° and the maximum range of motion of the shoulder elevation angle was 56°. The shoulder internal rotation had a range of motion of 36°. The maximum range of motion of the elbow flexion was 114°.

### 3.2. Synergy Extraction

**Figure 6:**
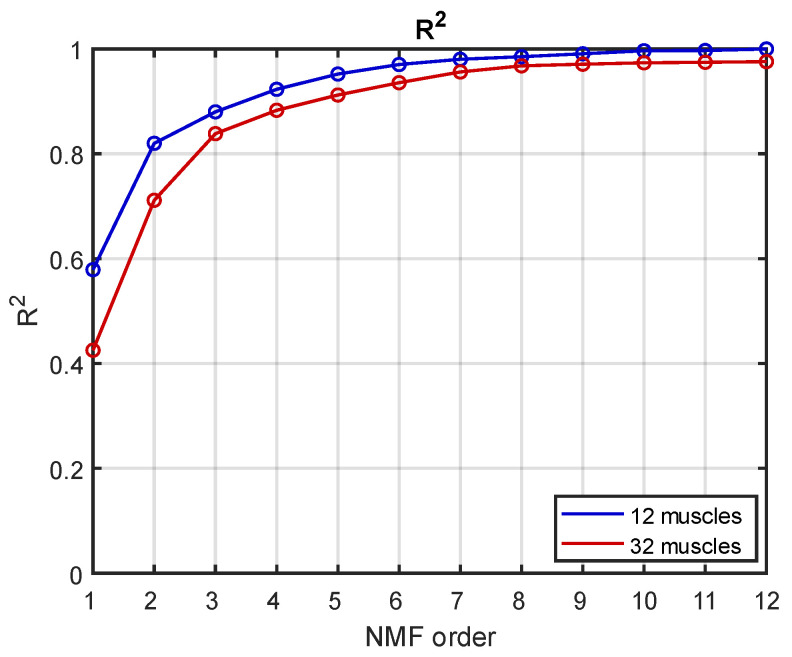
***R*^2^ comparison between the “standard” and the “high-density” datasets.** Comparison between the *R*^2^ obtained from the 12 muscles dataset (in blue) and the one obtained from the 32 muscles dataset (in red).

The goodness of reconstruction *R*^2^ is reported in Figure 6 for both the “standard” and the “high-density” datasets. With the “standard” dataset, the curve started at 0.58 and reached 1 at order 12; with the “high-density” dataset, the *R*^2^ was 0.42 at order 1 and 0.98 at order 12.

Considering the three threshold levels of reconstruction *R*^2^, the “high-density” dataset always needed an additional synergy with respect to the “standard” dataset, as reported in Table 1. Therefore, in the “high-density” muscle configuration, one extra synergy was needed to reach the same level of reconstruction of the signal.

**Figure 7:**
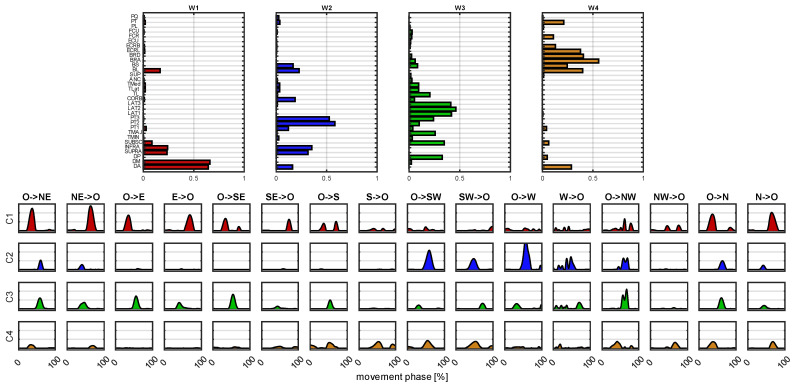
**Synergy structure and temporal coefficients.** Example of the synergies extracted from the 32 muscles dataset. The time-invariant components *W* are reported in the upper panel, while the corresponding coefficients *C* are reported in the lower panel.

In Figure 7, we reported an example of synergy extraction when four synergies were extracted, considering the “high-density” muscle configuration. The time-invariant components of the synergies W represent the muscle loads of the synergies, while the corresponding temporal coefficients C show the activations of the synergies in in each direction of motion.

**Figure 8:**
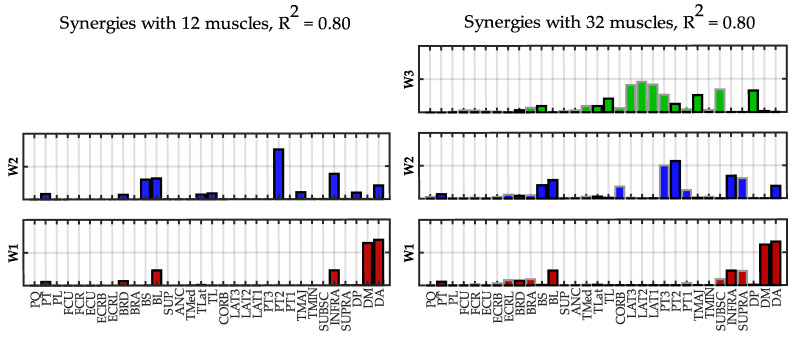
**Spatial synergies comparison (*R*^2^ = 0.80).** Spatial synergies extracted with *R*^2^ = 0.80 considering 12 muscles (**left column**) and 32 muscles (**right column**). The twenty additional muscle loads are depicted with a grey edge; the 12 muscles of the standard dataset are with a black edge.

In Figure 8, the synergies extracted at *R*^2^ = 0.80 using 12 and 32 muscles are compared.

The similarity between corresponding synergies was 1 for W1 and 0.98 for W2 (mean similarity = 0.99 ± 0.01). The similarity was very high and W1 and W2 had loads from the same muscles. The additional synergy W3 principally contained muscles not included in the “standard” dataset (latissimus dorsi and subscapularis) and the deltoid posterior and teres major. This synergy controls the adduction and flexion of the shoulder.

**Figure 9:**
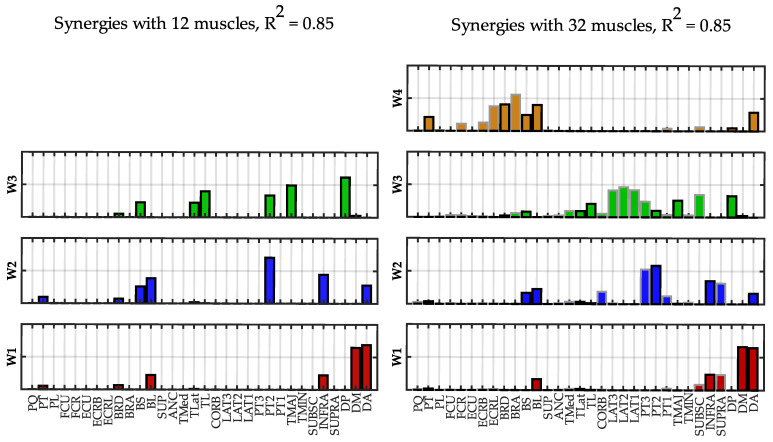
**Spatial synergies comparison (*R*^2^ = 0.85).** Spatial synergies extracted with *R*^2^ = 0.85 considering 12 muscles (**left column**) and 32 muscles (**right column**). The twenty additional muscles are depicted with a grey edge.

In Figure 9, the synergies extracted at *R*^2^ = 0.85 using 12 and 32 muscles are compared.

The similarity between corresponding synergies was 0.99 for all the synergies. W1 and W2 mainly contained muscle loads of the “standard” dataset, while additional muscles were added in W3, although the similarity of the shared muscles was very high. These muscles are the latissimus dorsi and the subscapularis that act with the triceps and the deltoid posterior for the flexion and adduction of the shoulder joint. The additional synergy W4 contains the biceps and the forearm muscles and is not captured with only 12 muscles.

**Figure 10:**
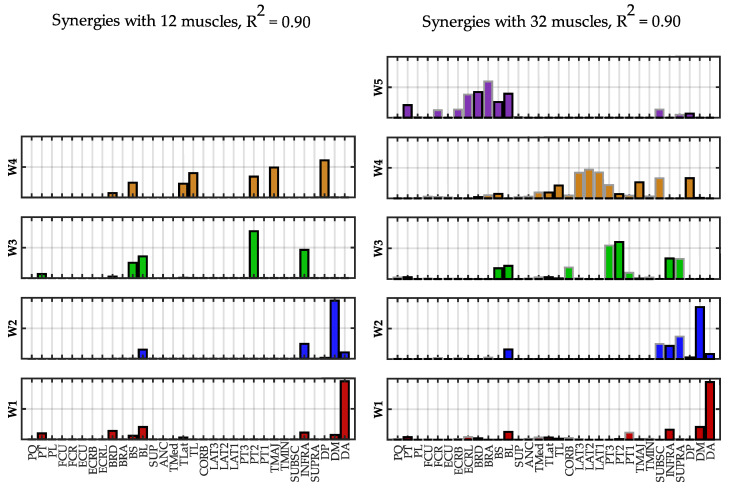
**Spatial synergies comparison (*R*^2^ = 0.90).** Spatial synergies extracted with *R*^2^ = 0.90 considering 12 muscles (**left column**) and 32 muscles (**right column**). The twenty additional muscles are depicted with a grey edge.

In Figure 10, the synergies extracted at *R*^2^ = 0.90 with 12 and 32 muscles are compared. The similarity between corresponding synergies was 0.98 for W1, 0.99 for W2 and W3 and 0.97 for W4 (mean similarity = 0.98 ± 0.01). The first three synergies mainly contain the same muscles of the “standard” dataset. W4 (32 muscles) is very similar to the W4 obtained with 12 muscles, adding the latissimus dorsi that has a similar function to the deltoid posterior and teres major. The additional synergy W5 contains muscles of the forearm and the biceps and was not identified using only 12 muscles.

### 3.3. Validation with Experimental Data

**Figure 11:**
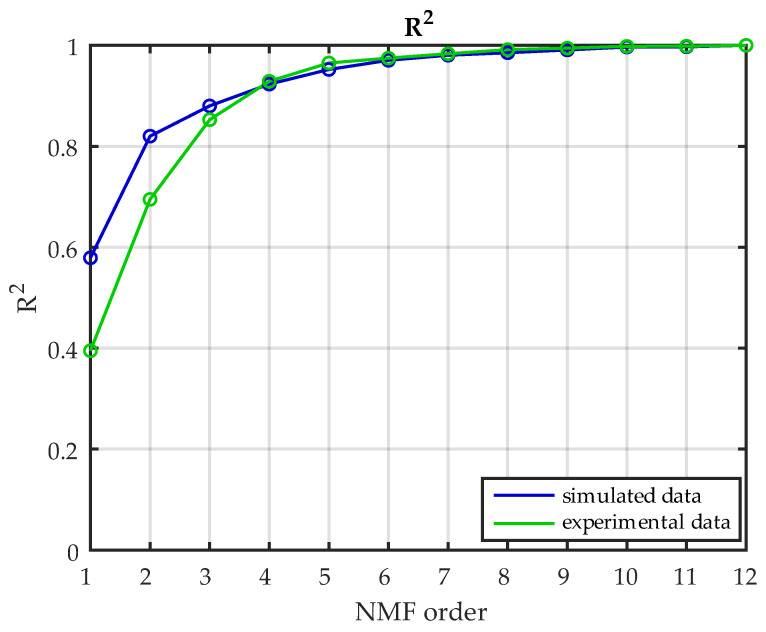
***R*^2^ comparison between simulated and experimental data.** Comparison between the *R*^2^ obtained from simulated data and the *R*^2^ obtained with experimental data.

The goodness of reconstruction *R*^2^ is reported for the experimental data and OpenSim ones (12 muscles) in Figure 11. The *R*^2^ with the simulated data was 0.58, while the *R*^2^ with the experimental data was 0.39 with one extracted synergy. Considering the threshold level *R^2^ =* 0.80, the experimental data needed three synergies, while the simulated data needed only two synergies. Both datasets needed three synergies when *R*^2^ = 0.85 and four synergies when *R*^2^ = 0.90.

**Figure 12:**
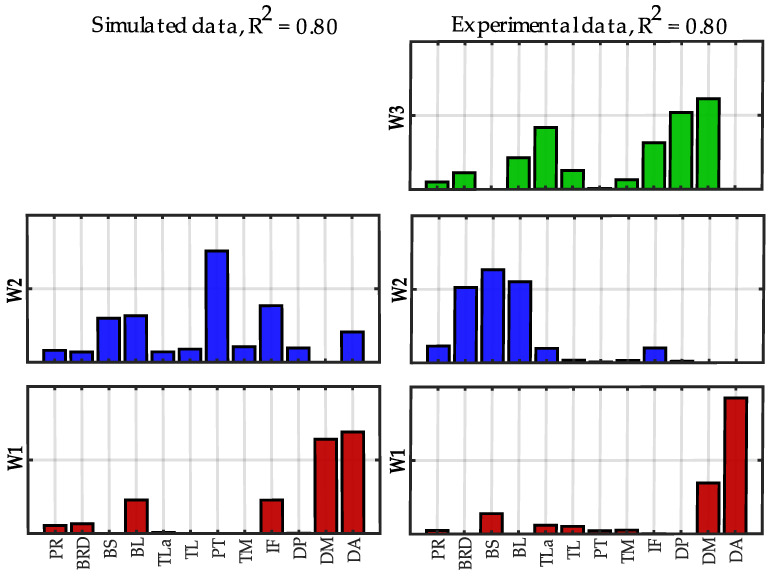
**Spatial synergies comparison at *R*^2^ = 0.80 between simulated and experimental data.** Spatial synergies extracted with *R*^2^ = 0.80 considering simulated data (**left column**) and experimental data (**right column**).

In Figure 12, the synergies extracted at *R*^2^ = 0.80 using simulated data and experimental data are compared.

The similarity between corresponding synergies was 0.86 for W1 and 0.46 for W2 (mean similarity = 0.66 ± 0.28). Synergy W1 was similar between the two conditions and involved the anterior and middle deltoid, typically used for elevating the shoulder. Synergy W2, instead, included the biceps in both the cases; however, in the simulated data, there was a shoulder contribution of the pectoralis and the infraspinatus, not found in the experimental data. Finally, the third synergy W3 in the experimental data showed the co-contraction of triceps and deltoids (middle and posterior) that was not identified in the simulated data.

**Figure 13:**
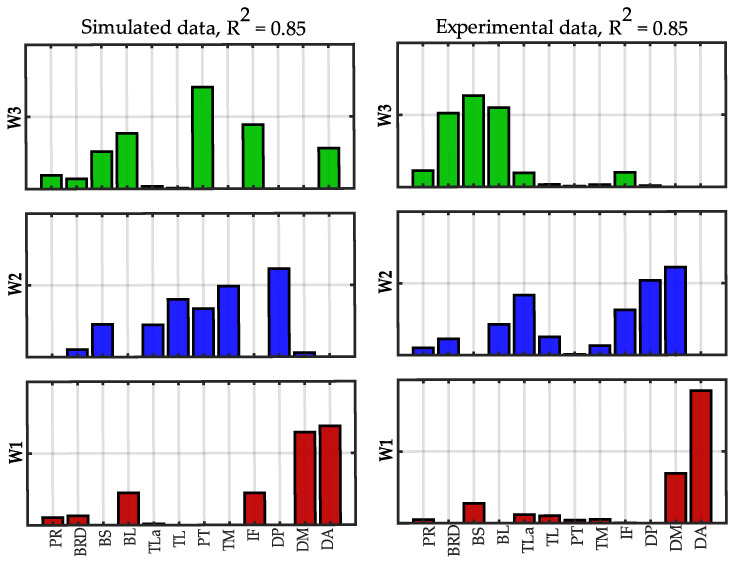
**Spatial synergies comparison at *R*^2^ = 0.85 between simulated and experimental data.** Spatial synergies extracted with *R*^2^ = 0.85 considering simulated data (**left column**) and experimental data (**right column**).

In Figure 13, the synergies extracted at *R*^2^ = 0.85 using simulated data and experimental data are compared.

The similarity between corresponding synergies was 0.86 for W1, 0.52 for W2 and 0.47 for W3 (mean similarity = 0.62 ± 0.21). Synergy W1 mainly involved the anterior and middle deltoid for the elevation of the shoulder and it was similar between the datasets. Synergy W2 included the triceps and the posterior deltoid in both the synergies. Instead, in the experimental data, there was also the contribution of the middle deltoid and infraspinatus, while the teres major and the pectoralis had small loads. Finally, the third synergy W3 in the experimental data showed the biceps and the brachioradialis, while in the simulated data, the pectoralis was also found.

**Figure 14:**
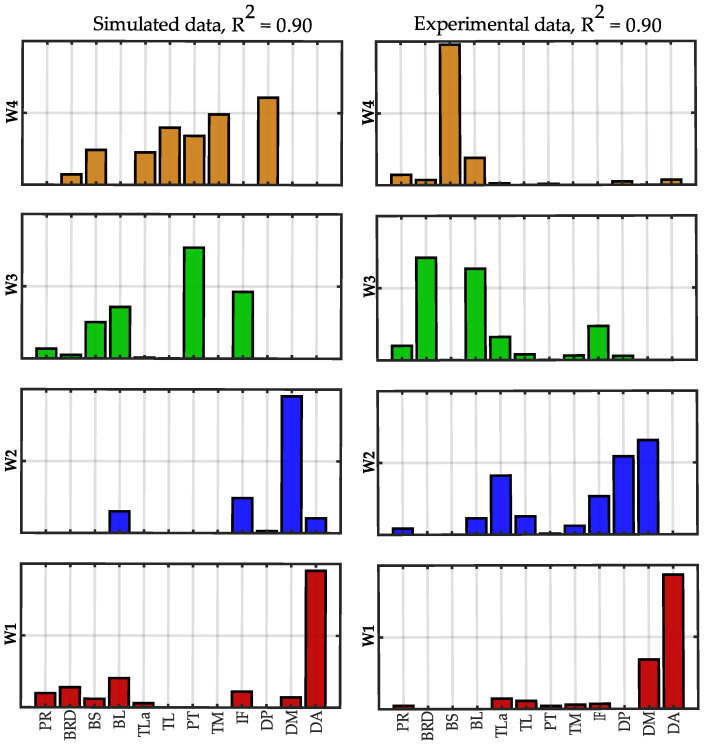
**Spatial synergies comparison at *R*^2^ = 0.90 between simulated and experimental data.** Spatial synergies extracted with *R*^2^ = 0.90 considering simulated data (**left column**) and experimental data (**right column**).

In Figure 14, the synergies extracted at *R*^2^ = 0.90 using simulated data and experimental data are compared. The similarity between corresponding synergies was 0.92 for W1, 0.71 for W2, 0.36 for W3 and 0.26 for W4 (mean similarity = 0.56 ± 0.31). Synergy W1 had a load of the anterior deltoid and had a higher similarity between the two conditions. Synergy W2 mainly involved the middle deltoid and, for the experimental data, it also included the posterior deltoid and the triceps. In the experimental data, the synergy W3 data showed the biceps long head and the brachioradialis, while in the simulated data the biceps were associated with the pectoralis. Finally, synergy W4 included the biceps short head in the experimental data and the triceps, the pectoralis and the infraspinatus in the simulated data.

## 4. Discussion

In this study, the synergies extracted from a standard EMG configuration (12 muscles) and from a “high density” one (32 muscles) of the upper limb during multi-joint movements were compared in terms of number and structure. Since technical and logistic issues limit the possibility of recording high-density EMG data in many practical scenarios, muscle activations were obtained using an OpenSim upper limb model, starting from experimental kinematic data. The goodness of reconstruction *R*^2^ was computed in both the 12 and 32 muscle configurations and muscle synergies were extracted with three minimal reconstruction *R*^2^ threshold levels—0.80, 0.85, 0.90. For each threshold, an additional synergy was extracted when using the high-density configuration, with respect to the standard dataset composed of 12 muscles. Finally, we compared the synergies extracted from the simulated data to the ones obtained from experimental EMG data.

The shared synergies between 12 and 32 muscles were very similar in terms of structure, showing a similarity higher than 0.97. In general, some muscles with the same function were added to existing synergies and complemented their structure (for example, latissimus dorsi was added to a synergy with the infraspinatus and posterior deltoid). The additional synergies in the high-density configuration, instead, mainly collected muscles of the forearm coupled with the biceps. Therefore, some additional muscles were added to existing synergies and the added muscles had similar functions and further specified the same physiological functions associated. On the contrary, the additional synergies underlay movements of the forearm and wrist that were not covered with the standard set of muscles. Steele et al. [23] already showed that both the number and the choice of muscles impact on synergy extraction, finding low similarity when using a small subset of muscles that increased when more than 10 muscles were considered.

The influence of the number of muscles considered in the synergy extraction could have an impact on clinical evaluations and motor control analysis. In fact, in some pathological conditions, muscle activity—especially the co-contraction of agonists and antagonists—is amplified or decreased (for example due to spasticity) and many muscles are activated at the same time in order to compensate for the reduced joint stiffness [34,35]. It follows that patterns of motor coordination are altered and the dimensionality of the control space could be identified more accurately with a complete mapping. Our results may also suggest an impact on the analysis of fine movements regarding forearm and wrist muscles, where muscle groups are small and located in overlapped layers. In an analysis based on a large set of hand grasps, one to three synergies were found with 12 electrodes for each subject [36] and three synergies were found with 15 to 19 muscles in primate grasping [37]. Therefore, our results are in accordance with previous studies that showed that the number of synergies may be underestimated when using relatively few EMG channels; this effect could be enhanced considering more subjects and movements, as demonstrated by Jarque-Bou et al. [38] in the kinematic domain. Movement is also influenced by the manipulation of objects with intention, that affects both kinematic and muscular patterns [39], revealing that neglected muscles, especially at the forearm level, are essential for quantifying motor control in purposeful movements and the impact of the dimensionality of the control space and structure of motor modules. Our results confirm that in a multi-directional reaching scenario in the sagittal plane, there is a moderate sub-estimation of the number of modules when a “low” threshold is chosen. At all the *R*^2^ levels, adding a fairly complete set of muscles determines the extraction of more synergies.

The use of a musculoskeletal model may be useful for muscle synergies when analyzing a large number of muscles that are difficult to record experimentally, due to technical issues such as location, risk of cross-talk and small muscle dimension, or due to set-up and time requirements. Typically, multiple muscles need to be recorded at the same time for a full evaluation of the motor performance of neurological patients [40,41]. Moreover, forearm and hand muscles are very difficult to record since they are small and located in multiple and deep layers; therefore, the musculoskeletal model can be used for multiple purposes such as the analysis of hand and wrist muscle synergies for hand motor control studies [33,36,42] and for the development of devices based on myoelectric control [43,44].

Comparing the results obtained from simulated and experimental data, we found that the same number of synergies were needed to reconstruct the same *R*^2^ level, except at *R*^2^ = 0.80 in which the experimental data needed an additional synergy. When comparing simulated and real data in the three levels of *R*^2^, the first synergy that mainly accounted for the anterior and middle deltoid had a high similarity, while the other synergies had only partially comparable structures. In particular, the synergies from the simulated data had a load associated to the pectoralis coupled with the biceps that was not found in the experimental data. Another difference was the component of the brachioradialis present in some synergies of the experimental data but not in the simulated data. Furthermore, the posterior deltoid in the simulated data was not found in the same synergies with the anterior and middle deltoids, as in the experimental data.

We also noted that when *R*^2^ = 0.80, the simulated data required only two synergies for EMG reconstruction. However, two generators are not enough to span a 2D multi-directional space [11] because at least three well-spaced synergies are needed to span such a domain. Interestingly, three synergies are extracted in the experimental data at the same reconstruction level. This probably indicates that in our study, thresholds higher than 0.80 were more appropriate to extract synergies from the OpenSim data. Moreover, in our scenario, the optimization procedure seemed to underestimate the antagonist muscle activity, generating mainly agonist-based synergies. For example, in phases of the movement in which the agonist muscle was the posterior deltoid, the anterior and middle deltoids should be partially active for the stabilization of the movement; instead, the simulation results showed that when the posterior deltoid was highly activated, the other deltoids were not activated. Similar considerations were reported by Kian et al. [45] when analyzing the activity of muscles needed for stabilizing the glenohumeral joint in OpenSim. These considerations come from a pilot study and more structured investigations should be conducted to verify whether our results can be applied to other scenarios. Mismatched muscle activations with respect to EMG data were also found in other studies regarding the lower limbs, where low to moderate correlations were reported between simulated and experimental muscle activity during walking [46].

In this study, only the 12 muscles dataset was compared with experimental data since the recording of numerous muscles could be difficult to realize. In future works, a comparison of the synergies extracted from 32 (or more) experimentally recorded EMG signals with the simulated data achieved with the 32 muscles model may be investigated. Since devices with 32 EMG probes are not usually available in laboratories, a suggested approach is to perform multiple recordings, only relocating some probes while keeping some channels fixed to guarantee and check the repeatability between multiple repetitions that are needed to acquire signals from 32 muscles.

The differences with respect to experimental data may also be improved with a better tuning of reserve actuators or with the choice of another model since other studies found high correlations between experimental EMG data and simulated activations in the lower limbs [47]. Future developments could be to include more subjects in the analysis in order to extend the results on a more numerous group and movement variability.

## 5. Conclusions

In this study, we investigated the difference found in the extracted muscle synergies when considering 32 muscles with respect to a typical standard protocol with 12 muscles from muscle activations simulated in OpenSim. Synergies extracted from 32 muscles presented an additional synergy that could not be identified with only 12 muscles. Therefore, the use of a small number of muscles may underestimate the dimensionality of motor control, producing effects on laboratory and clinical applications. The pilot results of this study should be confirmed with high-density experimental data.

## Figures and Tables

**Table 1:** Order of factorization needed to achieve the three threshold levels of reconstruction *R*^2^ with the “standard” and “high-density” dataset.

Order of Factorization			
	*R*^2^ = 0.80	*R*^2^ = 0.85	*R*^2^ = 0.90
12 muscles (standard)	2	3	4
32 muscles (high-density)	3	4	5

## Data Availability

The data presented in this study are available on reasonable request from the corresponding author.
